# Norwogonin Attenuates Inflammatory Osteolysis and Collagen‐Induced Arthritis via Modulating Redox Signalling and Calcium Oscillations

**DOI:** 10.1111/jcmm.70492

**Published:** 2025-03-18

**Authors:** Haojue Wang, Tao Yuan, Xiao Yu, Yi Wang, Changxing Liu, Ziqing Li, Shui Sun

**Affiliations:** ^1^ Department of Joint Surgery Shandong Provincial Hospital, Cheeloo College of Medicine, Shandong University Jinan Shandong China; ^2^ Department of Joint Surgery Shandong Provincial Hospital Affiliated to Shandong First Medical University Jinan Shandong China; ^3^ Department of Obstetrics and Gynecology Jian Gong Hospital Beijing China; ^4^ Orthopaedic Research Laboratory Medical Science and Technology Innovation Center, Shandong First Medical University & Shandong Academy of Medical Sciences Jinan Shandong China

**Keywords:** calcium oscillations, ERp57, Norwogonin, osteoclast, rheumatoid arthritis, ROS

## Abstract

Norwogonin is a flavonoid extraction derived from *Scutellaria baicalensis*. However, its potential mechanisms in the context of rheumatoid arthritis (RA) are unclear. This study investigates the specific effects and associated targets of Norwogonin in RA‐related inflammatory osteolysis. Network pharmacology was conducted to analyse the core targets and signalling pathways of Norwogonin in RA. In vitro experiments were carried out to explore the actual effects of Norwogonin on osteoclast behaviours and related signalling mechanisms. In vivo studies further validated the therapeutic effect of Norwogonin in collagen‐induced arthritis (CIA) mice. The network pharmacological analysis identified 18 shared targets between Norwogonin and RA, indicating a connection with inflammatory response and oxidoreductase activity. For biological validations, the results of in vitro experiments revealed 160 μM of Norwogonin inhibited LPS‐driven osteoclast differentiation and function. The qPCR assay and Western blot analysis also disclosed consistently diminished changes to osteoclastic marker genes and proteins due to Norwogonin treatment, including those for osteoclast differentiation (Traf6, Tnfrsf11a and Nfatc1), fusion (Atp6v0d2, Dcstamp and Ocstamp) and function (Mmp9, Ctsk and Acp5). Further mechanism study revealed Norwogonin suppressed LPS‐driven ROS production and calcium (Ca^2+^) oscillations. Also, intraperitoneal injection of 30 mg/kg Norwogonin every other day successfully mitigated clinical arthritis progression and attenuated bone destruction in the CIA model. Our study scrutinises Norwogonin's therapeutic prospects in treating RA and illustrates its inhibitory effects and potential mechanism within LPS‐induced osteoclastogenesis and CIA mice, providing a basis for further translational research on Norwogonin in the treatment of RA‐related inflammatory osteolysis.

## 1 Introduction

Rheumatoid arthritis (RA) is a prevalent, chronic and systemic autoimmune disease characterised by irreversible impairment of multiple small joints [1]. While the pathophysiology of RA remains relatively unclear, numerous risk factors have been identified, including female sex, hormonal influence, smoking and genetic predispositions [1]. Currently, the incidence of RA is on the rise, affecting about 20 million people worldwide, with an experience of 50% higher risk of cardiovascular mortality in RA patients [2]. Great progress has been made in early RA diagnostics and etiological studies, allowing for personalised and symptom‐modified interventions for RA patients [3]. Despite being accompanied by various extra‐articular symptoms, such as cardiovascular, pulmonary, neurological, gastrointestinal and renal comorbidities [2], the most prominent and concerning feature of RA remains the articular pathological changes [4]. The formation of the RA microenvironment is the result of a complex interplay among various immune and effector cells, including macrophages, B cells, T cells, osteoclasts and synovial cells. Briefly, the autoantigen production and T cell activation initiate an immune response and inflammatory reaction [5]. The infiltrating immune cells subsequently release inflammatory factors such as IL‐1β, IL‐17, as well as TNF‐α [6, 7], and interact with local resident cells, resulting in tissue destruction and pathological changes [8]. Presently, western medicine primarily utilises disease‐modifying antirheumatic drugs (DMARDs), non‐steroidal anti‐inflammatory drugs (NSAIDs) and corticosteroids to treat RA [9]. However, the subpar targeting capabilities and numerous side effects of these approaches urge the development of newer therapeutics [10, 11, 12]. For instance, Methotrexate, which is the first‐line treatment option for RA, has been limited in application due to various adverse events [13, 14, 15], particularly elevating the risk of severe detrimental outcomes in older patients with chronic kidney disease [16].

The escalated inflammatory bone erosion and para‐articular osteoporosis mediated by hyperactivated osteoclasts greatly contribute to the articular deformities and disabilities in RA patients. Reactive Oxygen Species (ROS) are widely recognised as a pivotal secondary messenger of osteoclastogenesis, driving the downstream signal transduction of the Receptor Activator of Nuclear Factor Kappa‐B Ligand (RANKL) signalling pathway and playing a central role as a mediator in inflammatory disorders [17, 18]. Lipopolysaccharide (LPS), a primary component of gram‐negative bacteria, is documented to simulate the inflammatory condition associated with RA. It has been demonstrated that the stimulation of osteoclast differentiation and function is achieved through increased ROS production and the activation of various inflammatory‐related factors, including tumour necrosis factor‐α (TNF‐α), interleukin‐1 (IL‐1) and IL‐6 [19, 20, 21, 22]. Moreover, a bi‐directional regulation exists between two primary regulating networks of ROS‐related signalling and Ca^2+^‐related signalling. Briefly, such inflammatory factors could further arouse the calcium oscillation and promote osteoclast differentiation [23], and the generation of ROS is dominated by NOX family protein expression under Ca^2+^ regulation [24].

Consequentially, biological agents that inhibit osteoclast activity, such as cytokine blockers that correspond to the aforementioned inflammatory factors, have shown improved targeting capacity and therapeutic efficacy in RA treatment [3]. Nevertheless, these treatments also encountered challenges; for example, a ceiling effect is observed, whereby only 10% to 20% of RA patients exhibit symptom remissions; long‐term use can lead to severe infections, and older patients may exhibit poorer efficacy and safety outcomes [10, 11, 12, 25]. Therefore, the quest continues for more efficient, safer and cost‐effective therapeutic agents to alleviate joint‐related symptoms in RA. Over the years, traditional Chinese medicine (TCM) and its derivatives have proven effective in both clinical practice and experimental validation of RA [26]. Notably, formulas such as Soufeng sanjie, Guizhi shaoyao zhimu decoction and Simiao pill, which comprise Saposhnikoviae radix (from Saposhnikovia divaricata (Turcz.) Schischk.), Cinnamomi ramulus (from *Cinnamomum cassia* (L.) D. Don), Radix clematidis (from Clematis chinensis Osbeck) and Anemarrhenae rhizome (from Anemarrhena asphodeloides Bunge), have all been found to attenuate inflammation, bone destruction, immune imbalance, cartilage degeneration and synovial hyperplasia in RA [27]. Bioactive compounds extracted from herbs used in the formula have been confirmed to play the real therapeutic role, including anti‐inflammatory, antioxidant, antibacterial and antidiabetic abilities [28]. Given that chronic inflammatory conditions and oxidative stress are common features of RA, natural compounds that possess anti‐inflammatory and antioxidant properties may exert corresponding therapeutic effects.

Flavonoid compounds, predominantly found in dietary plants or herbal medicines, have shown a range of health‐promoting benefits [29]. They also provide anti‐osteoclastogenic, antioxidant, anti‐inflammatory, immunomodulatory and cartilage protection effects in RA patients [30]. Containing abundant flavonoid compounds [31], *Scutellaria baicalensis* demonstrates excellent therapeutic effects during RA treatment [32]. However, the exact therapeutic component of *S. baicalensis* is yet undefined, and whether it will affect osteoclast biology is unknown. Norwogonin is a flavonoid compound extracted from *S. baicalensis*, which is used in the treatment of cancer, diabetes mellitus and infectious diseases [33, 34, 35]. Theoretically, it holds promise for RA treatment, although its effect and molecular mechanisms have not been extensively reported in prior studies. Owing to substantial advancements in big data and bioinformatics methodologies over the last decade, network pharmacology now enables a systematic and comprehensive exploration of multi‐drug, multi‐pathway and multi‐disease frameworks simultaneously [36]. By utilising methods such as target prediction from databases, protein–protein interaction (PPI) networks, Gene Ontology (GO), Kyoto Encyclopedia of Genes and Genomes (KEGG) enrichment analyses and molecular docking, network pharmacology can serve as a valuable instrument for analysing potential bioactive compounds from TCM or predicting drug targets and their related molecular mechanisms. Consequently, network pharmacology could be a useful tool for exploring the potential efficacy of Norwogonin in RA treatment.

Therefore, in this study, we employed network pharmacology to probe the potential relationship between Norwogonin and RA and to predict potential therapeutic targets and signalling pathways. Subsequent experimental validation was conducted based on the results from network pharmacology, aiming to explore the effect of Norwogonin on LPS‐induced osteoclast formation and function and validate its therapeutic role in a collagen‐induced arthritis model by alleviating oxidative stress and mitigating calcium signalling. This study tries to reveal the therapeutic potential and mechanism of Norwogonin, a major bioactive compound found in *S. baicalensis*, in alleviating inflammatory osteolysis under RA conditions.

## 2 Methods and Materials

### 2.1 Network Pharmacological Analysis

#### 2.1.1 Bioactive Compounds in *Scutellaria Baicalensis*

All components of *S. baicalensis* were obtained from the Traditional Chinese Medicine Systems Pharmacology Database and Analysis Platform (TCMSP) (http://lsp.nwu.edu.cn/tcmsp.php) [37]. Bioactive compounds were screened according to relevant parameters that reflect the percentage of orally administered drug entering systemic circulation (oral bioavailability, OB), the evaluation of drug‐likeness (drug‐likeness, DL) and Fractional water accessible surface area of all atoms with negative partial charge (FASA‐), the permeability of compound to intestinal epithelium (Caco‐2 permeability) and the permeability of compound to blood brain barrier (BBB) [37].

#### 2.1.2 Potential Targets of Norwogonin and RA

The potential targets of Norwogonin were obtained from databases including TCMSP, HERB (http://herb.ac.cn/) and SwissTarget (http://swisstargetprediction.ch/) databases. The disease targets related to RA were extracted from the DisGeNET database (http://www.disgenet.org/web/DisGeNET/menu/home) and the Comparative Toxicogenomics Database (CTD) (http://ctdbase.org/). Overlapping targets of Norwogonin and RA were then screened out of the UniProt database (https://www.UniProt.org/) [38] with the term ‘ *Homo sapiens* ’ as a filter.

#### 2.1.3 Protein–Protein Interaction (PPI) Network and Enrichment Analysis

To construct the PPI network, shared gene targets of Norwogonin and RA were inputted into the STRING online database (https://string‐db.org/) [39]. By defining organisms as ‘ *Homo sapiens* ’ and confidence as 0.4 (medium confidence), the resulting PPI network was established. Gene Ontology (GO) and Kyoto Encyclopedia of Genes and Genomes (KEGG) analyses were performed using the Database for Annotation, Visualisation and Integrated Discovery (DAVID) (https://david.ncifcrf.gov/home.jsp) [40], restricting the species to ‘ *Homo sapiens* ’ Terms with a *p*‐value below 0.05 were included only and subjected to further analysis. The Bioinformatics platform (https://www.bioinformatics.com.cn/) was then used to facilitate the visualisation of the GO biological process, KEGG pathways and interrelationships among targets and terms.

#### 2.1.4 Molecular Docking

The molecular structure of Norwogonin was retrieved from PubChem (https://pubchem.ncbi.nlm.nih.gov/) [41]. The three‐dimensional structures of the target proteins, including PIK3CG (2A4Z), IKBKB (3BRV), GSK3B (6TCU), PTGS2 (5F19) and ERp57 (7QPD) were acquired from the Protein Data Bank (PDB) database (https://www.rcsb.org/) [42]. Molecular docking between Norwogonin and these protein receptors was conducted using the MOE (version 2022) software. Preparation of the protein receptor included the removal of water and original ligand sequences, as well as using the ‘quick prep’ option in MOE. Preparation of the Norwogonin ligand included using the ‘wash’ and ‘energy minimise’ functions in MOE. An induction fitting mode was applied to all atoms while docking. All relevant data for the pharmacological analysis was obtained from the specified databases, which were last updated on October 10, 2023.

### 2.2 Reagents and Materials

Reagents for cell culture, including minimum essential medium α (α‐MEM, C12571500BT, Gibco), fetal bovine serum (FBS, 10099‐141C, Gibco) and penicillin/streptomycin (P/S, 15140122, Gibco), were all obtained from Gibco (Gaithersburg, MD, USA). Cytokines or chemicals used during osteoclast differentiation include macrophage colony‐stimulating factor (M‐CSF, 576406, Biolegend), receptor activator of nuclear factor‐κB ligand (RANKL, 769406, Biolegend), lipopolysaccharide (LPS) (L8274; Sigma‐Aldrich) and Norwogonin (Purity: ≥ 98%) (V34323, Invivochem). All primary antibodies employed for western blotting (WB) were derived from rabbit and procured from the following sources: anti‐CTSK (11239‐1‐AP) and anti‐Keap1 (10503‐2‐AP) were obtained from Proteintech (China); anti‐Nrf2 (A0674) and anti‐Nfatc1 (A1539) were obtained from ABclonal (China); ERp57 (2881S) was obtained from Cell Signaling Technology (USA). The secondary antibody used in this study was goat anti‐rabbit (SA00001‐2) procured from Proteintech (China).

### 2.3 In Vitro Cell Culture and LPS Stimulation

The process of cell culture was similar to our previous study [43]. In brief, bone marrow‐derived monocytes/macrophages (BMMs) were extracted from long bones of the hindlimb from 8‐to 12‐week‐old mice (C57BL/6). Isolated bone marrow cells were transferred to a complete medium (CM; α‐MEM containing 10% FBS and 1% P/S) and cultured for about 16 h. After lysing red blood cells, the collected nonadherent cells were incubated in CM with M‐CSF (20 ng/mL) at a density of 3 × 10^5^ cells/mL for 48 to 72 h until reaching a 30% ~ 50% confluence. BMMs were cultured with osteoclast differentiation induction medium (CM with M‐CSF (20 ng/mL) and RANKL (40 ng/mL)) for another 3 to 4 days to generate osteoclast precursors, and then RANKL was changed into LPS (150 ng/mL) to continually stimulate the process of osteoclast differentiation and function. For the treatment group, a corresponding concentration of Norwogonin was added to the CM from the onset of osteoclastogenesis induction.

### 2.4 Flow Cytometry

BMMs were collected as described in Part 2.3, with all procedures performed on ice. Cells were then incubated with TruStain FcX PLUS (anti‐mouse CD16/32) antibody (156,604, Biolegend) (1:200 dilution) for a blocking period of 10 min and subsequently treated with PE‐F4/80 antibody (123,110, Biolegend) (1:20 dilution) and APC‐CD11b antibody (101212, Biolegend) (1:80 dilution) for 30 min on ice. After rinsing with pre‐cold PBS of two times, Cytek Aurora (Cytek Biosciences, China) was utilised for flow cytometry, and the double‐positive cell rate was analysed using FlowJo software.

### 2.5 Cell Viability Assay

BMMs were cultured in a 96‐well plate at a density of 3 × 10^5^ cells/mL, and cellular toxicity of Norwogonin was detected by the Enhanced Cell Counting Kit 8 (CCK8, E‐CK‐A362, Elabscience) assay. Briefly, after different concentrations of Norwogonin (80, 100, 120, 140, 160 μM) were added to each well containing osteoclast differentiation induction medium for 48 h stimulation, CCK‐8 was added according to the manufacturer's instructions. Cells were then incubated at 37° for 2 h, and the absorbance at 450 nm wavelength was detected by a spectrophotometer (TECAN SPARK).

### 2.6 Tartrate‐Resistant Alkaline Phosphatase (TRAP) Staining

The TRAP staining solution was composed based on Dr. Chevalier's protocol with slight modifications [44]. In brief, the Acetate‐Tartrate buffer (AT buffer) was prepared in advance, composed of sodium acetate trihydrate (19 mg/mL), glacial acetic acid 100% (0.45%) and sodium tartrate trihydrate (150 μg/mL) in ultrapure water. Fast violet B salt (7 mg/mL) was later dissolved in the AT buffer and filtered through a 0.45 μm filter upon rigorous agitation. Naphthol solution was prepared with 2 mg/mL of naphthol AS‐TR phosphate disodium salt dissolved in AT buffer. The successful formation of the TRAP staining solution was indicated by the emergence of yellow precipitates upon fully blending equal volumes of the violet and naphthol solutions. Mature osteoclasts were then fixed with 4% paraformaldehyde (PFA) and stained with the TRAP solution. After a 2 h incubation at 37°, the cells were rinsed using a 4% sodium fluoride solution. The mature osteoclasts were identified and quantified based on their TRAP‐positive multinucleated nature with a minimum of three nuclei per well.

### 2.7 F‐Actin Phalloidin‐iFluor Staining

Mature osteoclasts were fixed with PFA for 15 min at room temperature, followed by permeabilisation and blocking that involved culturing with a solution containing 1% BSA‐PBST (1 g BSA and 0.25 mL Triton X‐100 in 100 mL PBS) for 10 min. Subsequently, cells were stained with a Phalloidin‐iFluor 594 buffer (ab176757, Abcam, 1:1000 diluted in 0.1% BSA‐PBS) at 37°C for 2 h in a dark environment. DAPI was then used to counterstain cells for nuclei identification. The cellular F‐actin rings and nuclei of cells were imaged using a three‐dimensional digital confocal system (EVOS M700, Thermo Fisher Scientific), and osteoclasts with or without F‐actin rings were counted separately to calculate the percentage of osteoclasts with intact F‐actin rings relative to the total number of osteoclasts.

### 2.8 Acridine Orange (AO) Staining

Mature osteoclasts were incubated with AO solution (A6014, Sigma‐Aldrich) as per the manufacturer's instructions at 37°C for 15 min. The stained acidic vesicles (red) and basic nuclei (green) were observed using a confocal imaging system (Celldiscoverer 7, Zeiss), and fluorescent intensity within the osteoclasts from the red and green channels was recorded and assessed by ZEISS ZEN 3.8 software. The intensity ratio of red/green was used to reflect the acid secretion function of osteoclasts.

### 2.9 Bone Resorption Assay

BMMs were seeded in a 48‐well plate coated with hydroxyapatite (CSR‐BRA‐48KIT; Cosmo Bio USA) with a density of 3 × 10^5^ cells/mL. The cell culture process was followed as introduced above in Part 2.3 with some modifications. Briefly, the replacement of RANKL with LPS was changed from Day 3 to Day 5, and LPS was employed for the osteoclastic induction in the last 3 days. On Day 8, the culture was suspended and rinsed with 5% sodium hypochlorite solution. After washing with ddH_2_O three times, the plate was dried under room temperature and observed with a light microscope. The ImageJ software was then employed to analyse and quantify the total resorption area of each well.

### 2.10 Intracellular ROS Measurement

Intracellular ROS level was determined using an H2DCFDA probe (S0033S, Beyotime) according to our previous publication [45]. Osteoclast precursor cells were cultured in the presence or absence of Norwogonin for 24 h and subsequently treated with the DCFH‐DA probe for 30 min at 37°C. Following the removal of the DCFH‐DA probe, RANKL (40 ng/mL), LPS (150 ng/mL) or a combination of LPS (150 ng/mL) and Norwogonin (160 μM) was introduced to each sample group. Upon undergoing stimulation for 10 min, the cells were examined via a three‐dimensional digital confocal system (EVOS M700, Thermo Fisher Scientific) and analysed utilising ZEISS ZEN 3.8 software.

### 2.11 Intracellular Ca^2+^ Oscillation Measurement

The determination of cellular Ca^2+^ oscillation was based on our previous publication [46]. Osteoclast precursors were treated with RANKL (40 ng/mL), LPS (150 ng/mL), or a combination of LPS (150 ng/mL) and Norwogonin (160 μM) for 3 h stimulation and incubated in CM containing 5 μM Fluo‐4 AM (20,552, AAT Bioquest) and 0.05% F127 (Sigma) for 40 min avoiding light. Cells were then rinsed with PBS and added to the above‐mentioned agents dissolved in isotonic solution (ISO; ddH_2_O as solvent, 105 mM NaCl, 5 mM KCl, 6 mM HEPES acid, 4 mM Na HEPES, 5 mM NaHCO_3_, 60 mM Mannitol, 5 mM D‐glucose, 1.3 mM CaCl_2_ and 0.5 mM MgCl_2_). The fluorescent images were captured at 5 s intervals lasting for 20 min. Ten cells were chosen for final analysis with ImageJ software, quantifying the fluorescence intensity at different times to indicate the Ca^2+^ oscillations.

### 2.12 Western Blotting (WB)

WB was performed as described in the previous publication with slight modifications [47]. Cells were collected and lysed in RIPA buffer (R0020, Solarbio) containing 1% phosphatase inhibitor (CW2383; Cwbio) and 1% protease inhibitor (CW2200; Cwbio). BCA Protein Assay Kit (PC0020, Solarbio) was then used to determine the protein concentration of each group according to the manufacturer's instructions. 30 μg denatured proteins and 5 μL prestained protein marker (WJ102, Epizyme) in each well were electrophoresed on an 8% SDS‐page gel and transferred to a polyvinylidene fluoride (PVDF) membrane (0.22 μm). The membrane was then blocked by protein‐free blocking buffer (PS108P, Epizyme) at room temperature for 1 h and incubated with primary antibodies (1:1000 diluted in 1%BSA‐TBST) at 4°C overnight. HRP‐conjugated anti‐rabbit secondary antibody (1:3000 diluted in 5% nonfat powdered milk‐TBST) was used to conjugate protein bands by incubating at room temperature for 2 h. The western ECL substrate (BioRAD) reacted blots were visualised by ChemiDoc touch imaging system (Bio‐Rad, Hercules, CA, USA) and analysed using the Image J software.

### 2.13 Quantitative Real‐Time Polymerase Chain Reaction (qPCR)

The total RNA of cells was extracted using TRizol agent (9109, TaKaRa). The reverse transcription of total RNA into cDNA was performed with an RT Premix kit (AG11706, Accurate Biology). Primers were firstly diluted to a storage solution of 100 μM and further diluted to a 10 μM concentration using a working buffer, as recommended in the manufacturer's protocol. Subsequent amplification and detection procedures were performed using the SYBR Green Premix kit (AG11701, Accurate Biology) and LightCycler 480II (Roche). GAPDH expression was used as the endogenous control to analyse the relative expression of Acp5, Atp6vod2, Ctsk, Dc‐stamp, Oc‐stamp, Mmp9, Nfatc1, Tnfrfs11a and Traf6 using a $2^{-\Delta \Delta C_{t}}$ method. All used primer sequences are shown in Table 1.

**TABLE 1:** Primer sequences for quantitative real‐time PCR.

Target (GenBank accession no.)	Primers
Acp5 (NM_001102405.1)	F: ACCTTGGCAACGTCTCTGCAC
	R: GTCCAGCATAAAGATGGCCACA
Atp6v0d2 (NM_175406.3)	F: AACTCAGCAGGACTATGTCAACC
	R: CTTCTTCCTCATCTCCGTGTCAAT
Dcstamp (NM_029422.4)	F: TTCTCGTGTCAGTCTCCTTCTACC
	R: TTTCCCGTCAGCCTCTCTCAA
Ocstamp (NM_029021.1)	F: GTTCTGGACTTCATCCTCTTCGT
	R: GTGGTTGAGCCTGTGGTAGAT
Mmp9 (NM_013599.5)	F: GCCCTGGAACTCACACGACA
	R: TTGGAAACTCACACGCCAGAAG
Tnfrsf11a (NM_009399.5)	F: GCTTACCTGCCCAGTCTCATC
	R: AAGCATCATTGACCCAATTCCAC
Traf6 (NM_001303273.1)	F: AAAGCGAGAGATTCTTTCCCTG
	R: ACTGGGGACAATTCACTAGAGC
Nfatc1 (XM_036161029.1)	F: CCGTTGCTTCCAGAAAATAACA
	R: TGTGGGATGTGAACTCGGAA
Ctsk (NM_007802.4)	F: CTTCCAATACGTGCAGCAGA
	R: TCTTCAGGGCTTTCTCGTTC

### 2.14 Collagen‐Induced Arthritis (CIA) Model

Eight‐week‐old DBA/1 male mice (Beijing Vital River Laboratory Animal Technology Co. Ltd., China) were randomly assigned into three groups (Control, CIA, CIA + Norwogonin). The mice in the CIA and CIA + Norwogonin groups received 100 μL CFA emulsion (containing 50 μL bovine type II collagen (2 mg mL^−1^, Chondrex, 20,022) and 50 μL Complete Freund's Adjuvant (Sigma‐aldrich)) from tail‐based subcutaneous injection on Day 0. And booster immunisation was performed on Day 21 with IFA emulsion (containing 50 μL bovine type II collagen and 50 μL Incomplete Freund's Adjuvant (Sigma‐aldrich)). From Day 24, mice in the CIA + Norwogonin group received 30 mg/kg Norwogonin through intraperitoneal (IP) injection every other day until Day 38. The arthritic score was observed and recorded from Day 24 to Day 40 based on a previous study [48]. The arthritic score criteria are presented as follows: 0 = no evidence of erythema or swelling; 1 = erythema and mild swelling confined to the tarsals or ankle joint; 2 = erythema and mild swelling extending from the ankle to the tarsals; 3 = erythema and moderate swelling extending from the ankle to metatarsal joints; and 4 = erythema and severe swelling encompassing the ankle, paw and digits, or ankylosis of the limb [49].

### 2.15 Micro‐Computed Tomography (Micro‐CT) Analysis

The hinder limbs of mice were extracted 16 days post the initial injection of Norwogonin and were subsequently fixed with 4% PFA for a 24 h period under 4°C, rinsing and storing with 70% ethanol afterwards. Both the paw and ankle joints were scanned utilising a Micro‐CT (Quantum GX2), with the parameters set at 90 KV, 88 μA, high resolution and 14 min duration. The three‐dimensional (3D) reconstruction was then performed to provide a top and lateral view of the bone structure.

### 2.16 Statistical Analysis

All experiments were performed as biologically independent quintuplicates. GraphPad Prism 9.4 software was used for data analysis, and results were presented as the mean ± standard deviation (SD). Either a T‐test or one‐way ANOVA was adopted for statistical comparisons between two groups or among multiple groups, respectively. Before all analyses, normality tests were undertaken. For two‐group comparisons, the Mann–Whitney test was applied for non‐normally distributed data, whereas an unpaired T‐test with or without Welch's correction was used depending on the equality of SD. For comparison among three groups, the Kruskal‐Wallis test was employed for non‐normally distributed data, while one‐way ANOVA or Welch ANOVA was performed for groups with identical or differing SD. A *p*‐value under 0.05 was considered statistical significance.

## 3 Results

### 3.1 Identification of Bioactive Components in *Scutellaria Baicalensis* and Its Targets on RA

**FIGURE 1:**
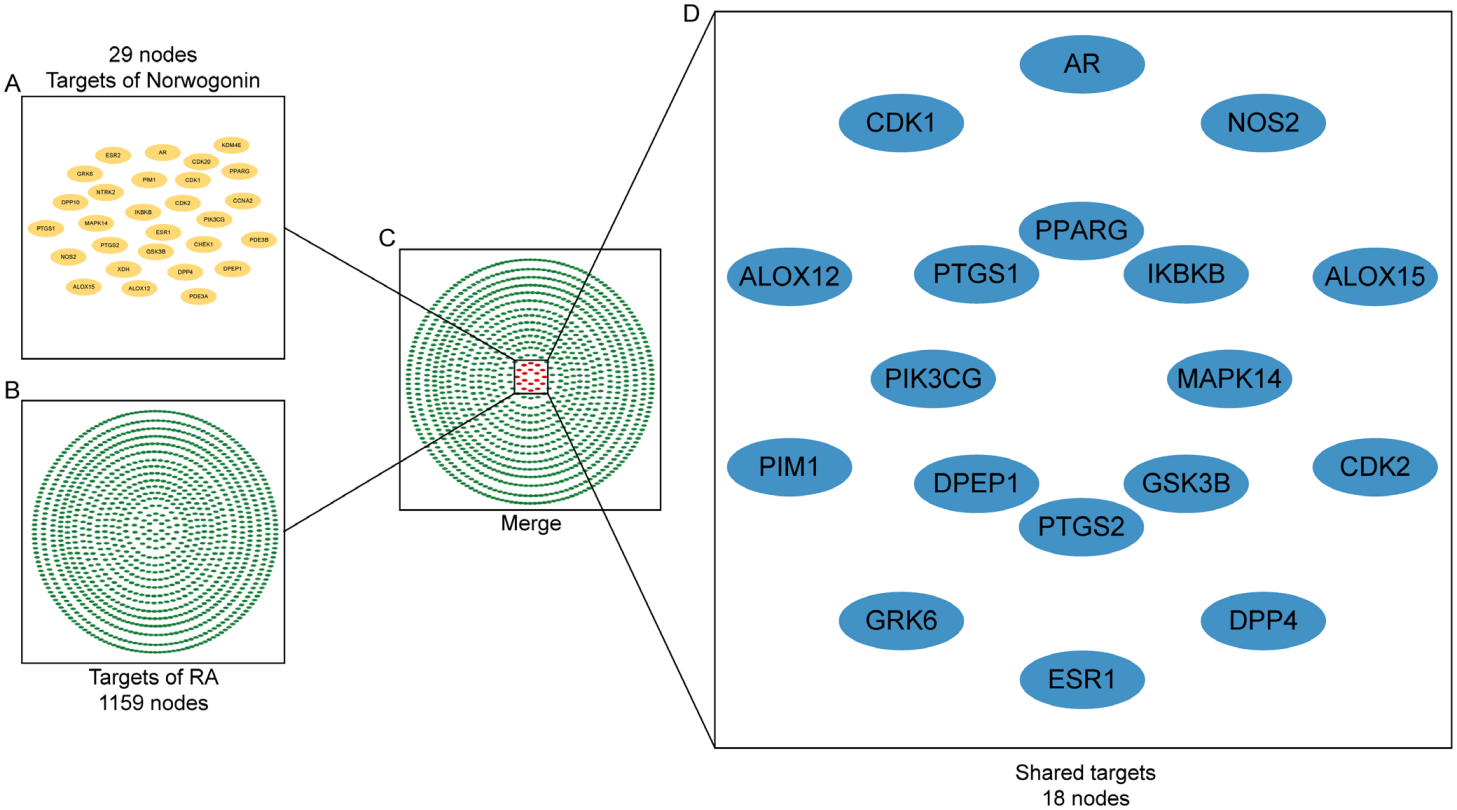
Identification of shared targets between Norwogonin and Rheumatoid arthritis (RA). (A) 29 predicted targets of Norwogonin. (B) 1159 potential targets associated with RA. (C) Circular diagram outlined the (D) overlapping gene targets related to Norwogonin and RA, highlighting 18 shared targets.

A total of 143 constituents of *S. baicalensis* were extracted from the TCMSP database as presented in Table S1. Upon the application of screening parameters including OB ≥ 30%, DL ≥ 0.18, Caco‐2 ≥ 0.5, FASA > 0.3 and BBB < 0, Norwogonin, along with two other bioactive compounds, were isolated, and a superior position was shown for Norwogonin when ranked by OB (See Table 2). Utilising TCMSP, HERB and SwissTarget databases (probability ≥ 40), 29 separated targets that related to Norwogonin were identified (Table S2). For the identification of RA‐related targets, the term ‘rheumatoid arthritis’ was employed for database searches in DisGeNET and CTD. Considering a relatively high amount of RA‐related targets in these databases, an inference score greater than 26 was set to filter the targets from CTD. This process identified a total of 1159 targets between the two databases as RA‐related targets (Table S3). Subsequently, the intersection of the results from both Norwogonin potential targets and RA‐related targets led to a total of 18 identical targets deemed as core genes for further use (Figure 1), especially employed in the functional analysis.

**TABLE 2:** Three chosen bioactive compounds from *Scutellaria baicalensis*.

Molecule name	MW	AlogP	Hdon	Hacc	OB	Caco2	BBB	DL	FASA	HL
Norwogonin	270.3	2.33	3	5	39.4	0.6	−0.17	0.21	0.39	16.93
Acacetin	284.3	2.59	2	5	34.97	0.67	−0.05	0.24	0.35	17.25
Baicalein	270.3	2.33	3	5	33.52	0.63	−0.05	0.21	0.36	16.25

### 3.2 Norwogonin‐RA Target Network and Analysis

**FIGURE 2:**
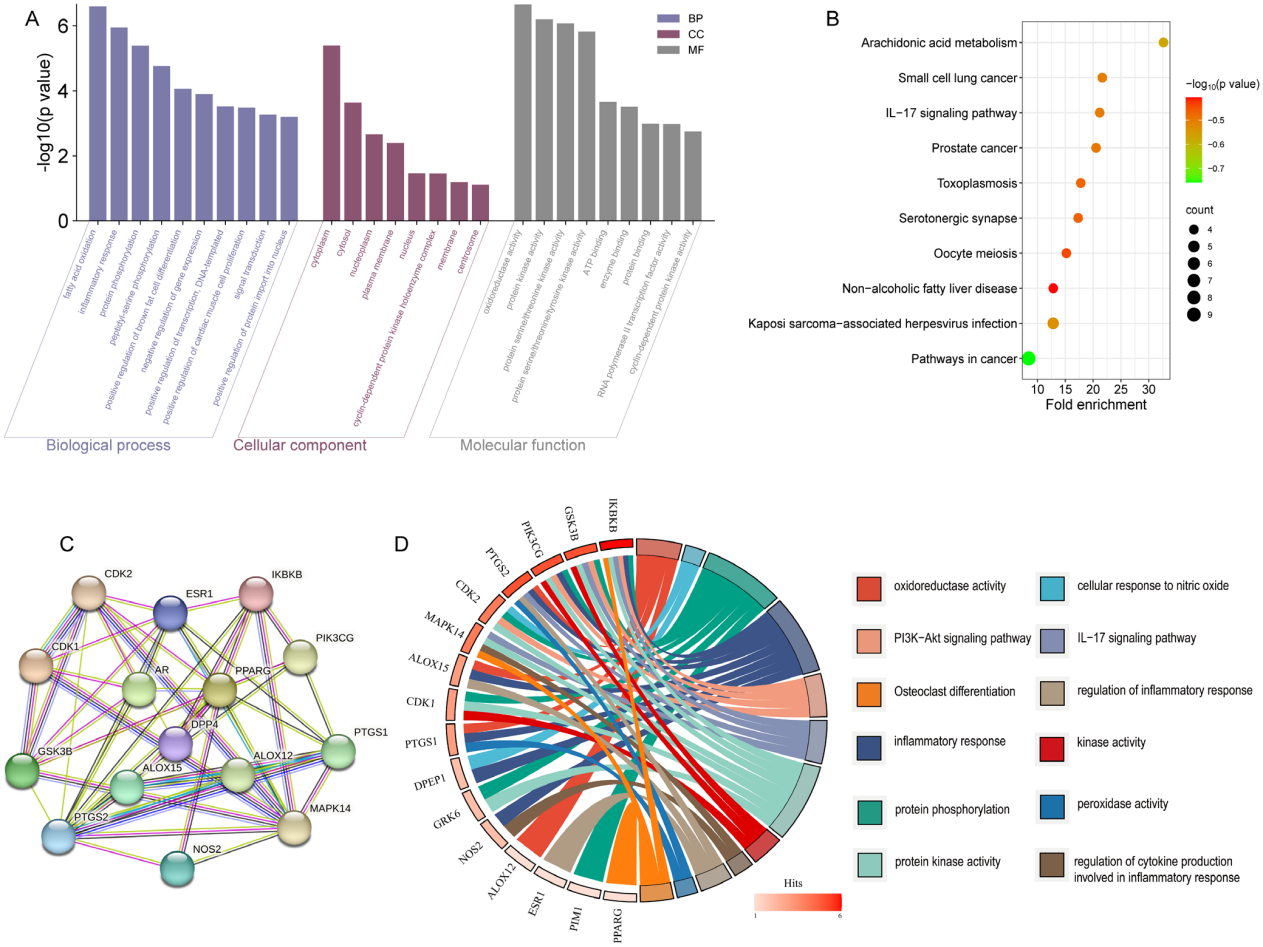
Norwogonin‐RA target network and analysis. (A) Histogram of Gene Ontology (GO) enrichment analysis showcases the top 10 significant terms of biological process, cell component, and molecular function according to the 18 shared targets. (B) Bubble chart of KEGG pathway analysis corresponding to the shared targets. The top 10 enriched pathways are shown. (C) Protein–Protein Interaction (PPI) and (D) chord diagram reflect the relationship network between putative targets and terms related to RA.

The GO and KEGG enrichment analyses were then conducted to explore the possible mechanisms of Norwogonin in the treatment of RA. By uploading 18 core genes to David (v6.8), a total of 121 GO and KEGG terms were significantly enriched (*p* < 0.05), including 52 biological process (BP) terms, 6 cellular component (CC) terms, 29 molecular function (MF) terms and 34 pathway terms (Table S4). The most significantly enriched terms for BP, CC, MF and KEGG are displayed (Figure 2A,B) and suggest that Norwogonin may interfere with the inflammatory process of RA via protein kinase activity and oxidoreductase activity in the cytoplasm, wherein the IL‐17 signalling pathway was involved. A PPI network was further retrieved from the STRING database using the same 18 core genes, during which the minimum interaction score was set at 0.4, and disconnected proteins were excluded (Table S5) (Figure 2C). The results implied that PTGS2, IKBKB and MAPK14 may act as hubs among these proteins. Moreover, their identification suggests that Norwogonin treats RA through a variety of potential pathways and targets. To further elucidate the connection between core targets and RA or osteoclast‐related terms, a target‐term interaction network was constructed and measured (Figure 2D). The above findings indicated multiple core targets of Norwogonin treatment, including IKBKB, GSK3B, PIK3CG and PTGS2.

### 3.3 Revelation of Potential Action Mode of Norwogonin in RA‐Related Targets

**FIGURE 3:**
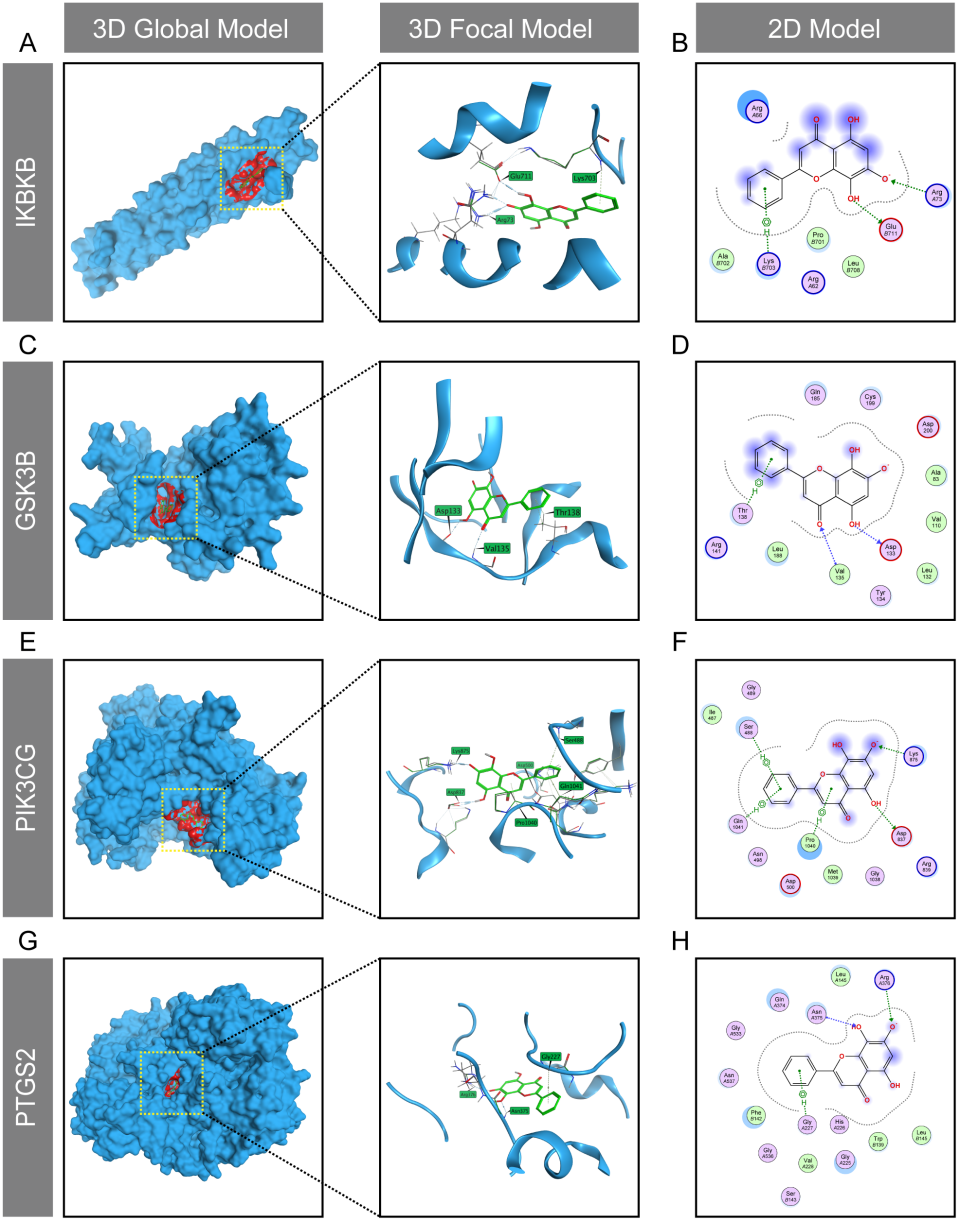
Revelation of the potential action mode of Norwogonin in RA‐related targets. The molecular docking mode of Norwogonin with IKBKB (A, B), GSK3B (C, D), PIK3CG (E, F) and PTGS2 (G, H) shown in 3D or 2D model.

To confirm the binding mode of Norwogonin with these potential core targets, molecular docking was conducted, primarily through ionic bonds, hydrogen (H) bonds or H–pi bond interactions. The results showed that Norwogonin could successfully bond with multiple sites of the targeted proteins, including ArgA73, LysB730 and GluB711 residues of IKBKB (Figure 3A,B), Val135, Thr138 and Asp133 residues of GSK3B (Figure 3C,D), Ser488, Lys875, Pro1040, Gln1041 and Asp837 residues of PIK3CG (Figure 3E,F), as well as ArgA376, AsnA375 and GlyA227 residues of PTGS2 (Figure 3G,H). The binding energy was subsequently calculated to reflect the affinity between the ligand and receptor. As illustrated in Table 3, the binding energies between Norwogonin and IKBKB, GSK3B, PIK3CG and PTGS2 are −5.5, −5.3, −5.6 and −6.2, respectively. These energy levels indicate a strong binding affinity and a high probability of these proteins interacting with Norwogonin. Serving as canonical pathways in both osteoclast differentiation and inflammatory reaction, the PI3K/AKT/GSK3β and NF‐κB signalling pathways significantly dominate the cellular behaviours of inflammatory osteoclasts [50, 51]. Additionally, PTGS2, also known as cyclooxygenase‐2 (COX2), emerges as a pivotal mediator during inflammation, specifically activated in LPS‐stimulated osteoclast precursor cells [52]. Consequently, our bioinformatics results imply a strong correlation between the therapeutic mechanisms of Norwogonin in RA through attenuating inflammatory osteolysis.

**TABLE 3:** Docking results of Norwogonin with key targets.

Target	Binding energy/(kcal/mol)	Binding residues
PTGS2	−6.2	Arg, Asn, Gly
PIK3CG	−5.6	Lys, Asp, Pro, Gln, Ser
IKBKB	−5.5	Arg, Glu, Lys
GSK3B	−5.3	Asp, Thr, Val

### 3.4 Norwogonin Impairs LPS or RANKL‐Induced Osteoclastogenesis

**FIGURE 4:**
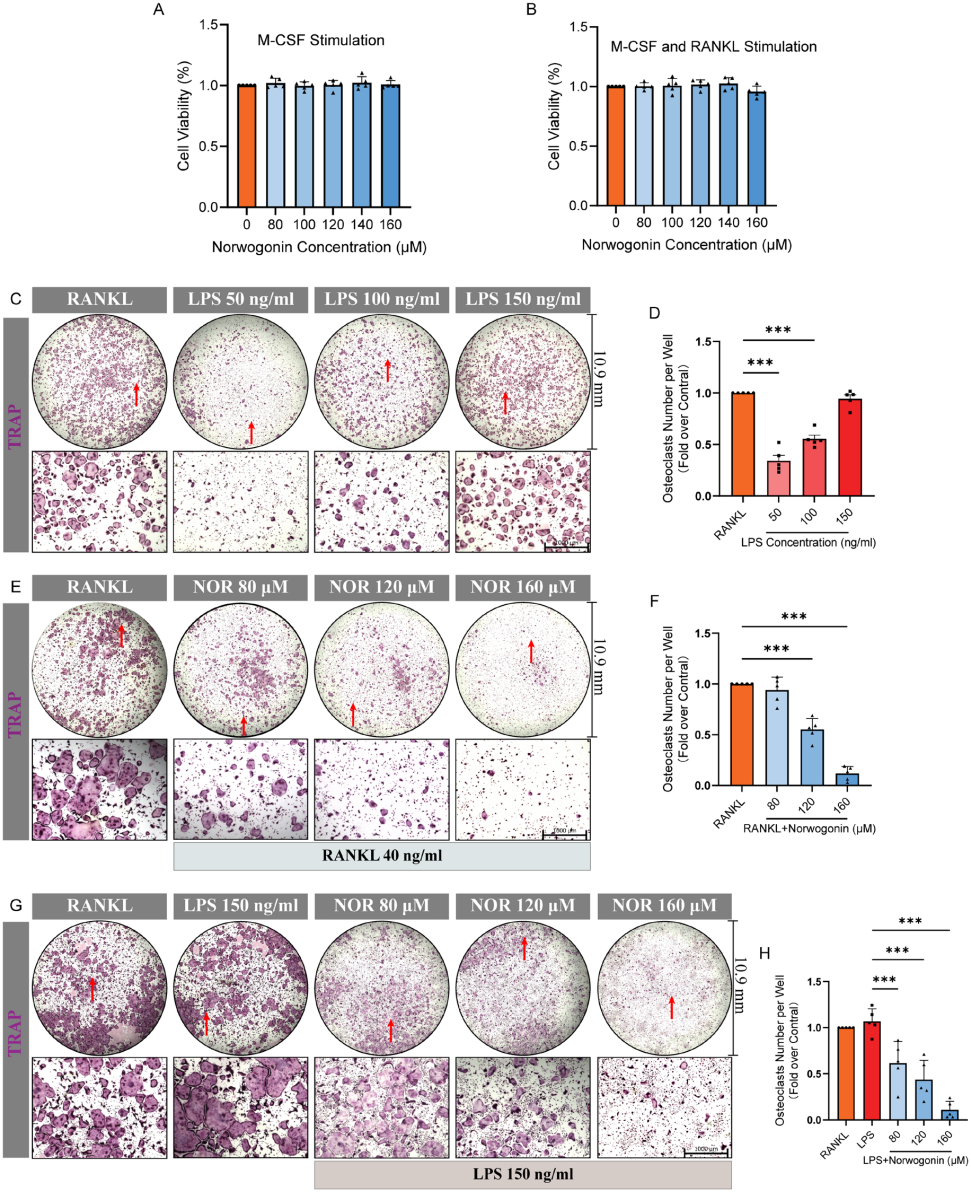
Norwogonin impairs LPS or RANKL‐induced osteoclastogenesis. (A, B) CCK‐8 assay for cell viability test. Bone marrow‐derived monocytes/macrophages (BMMs) were stimulated with Norwogonin (0, 80, 100, 120, 140 and 160 μM) in complete medium with M‐CSF (20 ng/mL) only (A) or with M‐CSF (20 ng/mL) and RANKL (40 ng/mL) (B) for a period of 48 h. (C) TRAP staining of osteoclasts under different concentrations of LPS. BMMs were stimulated with osteoclastogenesis induction medium (OIM) (comprising CM with M‐CSF and RANKL) for a 5‐day period for full differentiation, or initially pretreated with OIM for 60 ~ 72 h to generate adequate pre‐osteoclasts, after which the medium was replaced with M‐CSF and LPS (50, 100 and 150 ng/mL) for subsequent treatment. (D) Quantitative analysis of TRAP‐positive cells with more than three nucleus (TRAP+ MNCs) in (C). (E, G) TRAP staining of osteoclasts differentiated from BMMs under various treatments of Norwogonin. BMMs were induced with OIM for a full 5‐day osteoclast differentiation with different Norwogonin concentrations (0, 80, 120 and 160 μM), or initially stimulated with OIM and Norwogonin for a 60 ~ 72 h pretreatment to generate pre‐osteoclasts, after which the medium was replaced with M‐CSF and LPS (150 ng/mL) with Norwogonin, to induce the remaining differentiation. (F, H) Quantitative analysis of TRAP+ MNCs in (E) and (G). All quantitative data were analysed with T‐test or one‐way ANOVA, and presented as mean ± SD from five biologically independent experiments. ***p < 0.001, **p < 0.01, *p < 0.05. Red arrow indicates the position of microscope image in the whole‐well image. NOR: Norwogonin.

Given the established relevance of Norwogonin with osteoclast‐ and inflammation‐related pathways presented above, we selected LPS‐induced osteoclastogenesis, a classical in vitro model for inflammatory bone erosion in RA [53, 54], for subsequent biological validations. A flow cytometry analysis was performed, revealing that about 60% of cells isolated for osteoclast induction were macrophages (Figure S1). To evaluate the actual effect of Norwogonin on osteoclast differentiation under RA conditions, as hypothesised, we first conducted the CCK‐8 assay to determine the viability of osteoclast precursor cells under a 48‐h OIM stimulation (with or without RANKL) in the presence of various concentrations of Norwogonin (80, 100, 120, 140, 160 μM). The results demonstrated no cellular toxicity of Norwogonin within the specified concentrations (Figure 4A,B), thereby establishing a minimum biosafety border for subsequent biological tests.

Concurrently, to determine the appropriate dose of LPS for osteoclast induction, BMMs were initially pretreated with OIM (in the presence of RANKL) for a period of 60 to 72 h to generate sufficient pre‐osteoclasts, as previously described in a study [55]. Subsequently, RANKL was replaced with indicated concentrations of LPS (50, 100 and 150 ng/mL) for further cultivation. After 5 days of complete differentiation of osteoclasts, TRAP staining was carried out to identify qualified mature osteoclasts. For these RANKL‐pretreated pre‐osteoclasts, a dose‐dependent response to LPS stimulation was observed, with a concentration of 150 ng/mL LPS presenting a similar effect as the RANKL group (Figure 4C,D). Next, pre‐osteoclasts continuously stimulated with RANKL or replaced by LPS were treated with Norwogonin. Both groups demonstrated a dose‐dependent inhibitory effect, and a significant impediment in osteoclastogenesis was observed at a concentration of 160 μM, which was therefore chosen for the following experiments (Figure 4E–H).

### 3.5 Norwogonin Suppresses the Resorption Function of Osteoclasts

**FIGURE 5:**
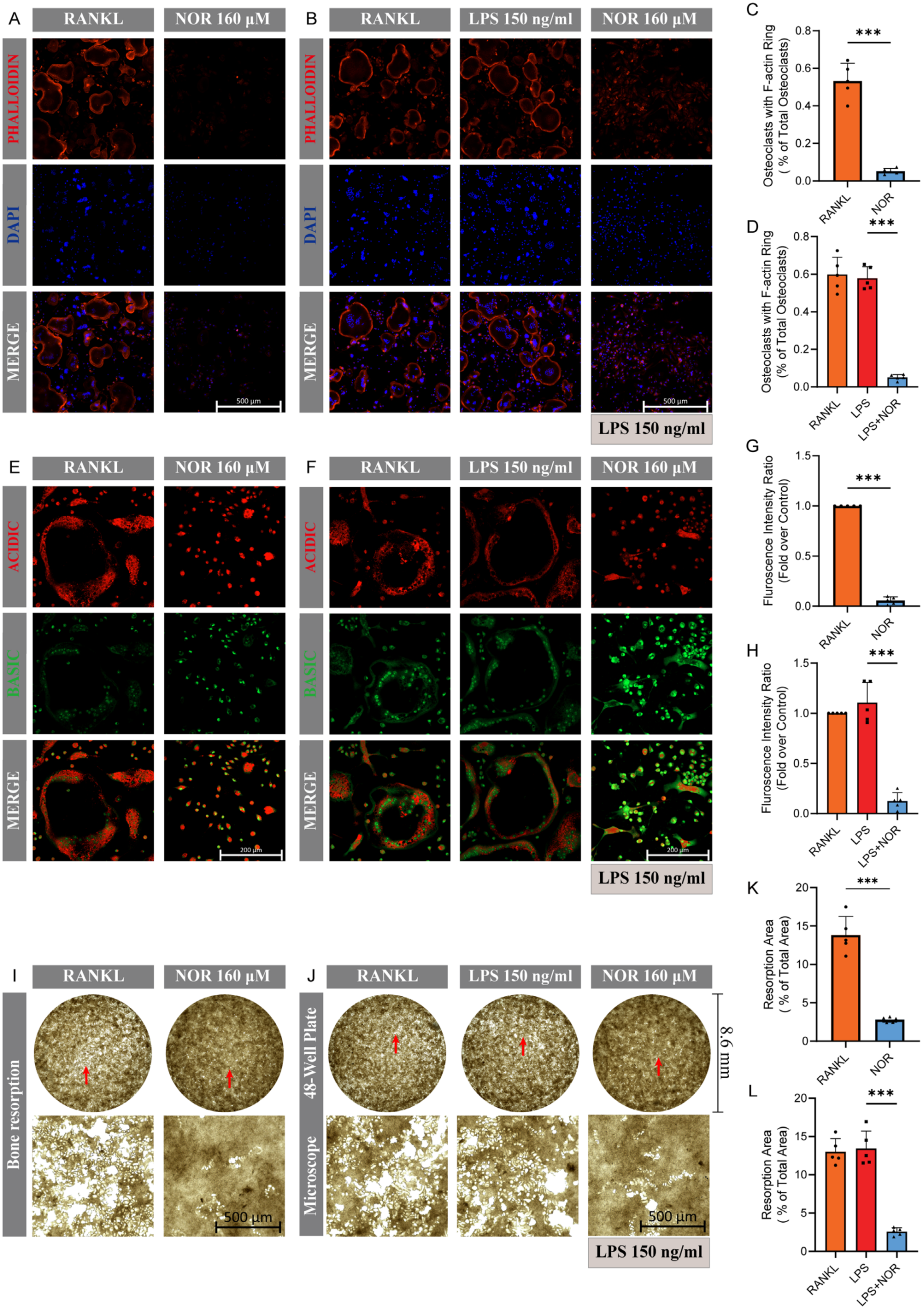
Norwogonin suppresses the resorption function of osteoclasts. (A, B) Effect of Norwogonin on F‐Actin ring formation. Cell culture was performed as previously described in Figure 4, and the F‐Actin rings (red) and nucleus (blue) were visualised under a confocal microscope after staining with phalloidin‐iflour 594 and DAPI. (C, D) Quantitative analysis of the proportion of osteoclasts displaying F‐Actin rings in (A) and (B). (E, F) Effect of Norwogonin on the acid secretion ability of osteoclasts. Acridine orange (AO) staining was performed to visualise the acidic vesicles (red) of osteoclasts. (G, H) Quantitative analysis of the fluorescence intensity ratio (red to green) of osteoclasts as shown in (E) and (F). (I, J) Effect of Norwogonin on the bone resorption function of osteoclast. (K, L) Percentage of bone resorption area in (I) and (J). All quantitative data were analysed with T‐test or one‐way ANOVA, and presented as mean ± SD from five biologically independent experiments. Red arrow indicates the position of microscope image in the whole‐well image. ***p < 0.001, **p < 0.01, *p < 0.05. NOR, Norwogonin.

We next examined the inhibitory effect of Norwogonin on osteoclast function. The formation of F‐actin rings is a prerequisite for osteoclasts to exert resorption function [47]. Via the visualisation of phalloidin staining, we discerned intact F‐actin rings in approximately 50% of the osteoclasts in either the RANKL or LPS groups. In contrast, the Norwogonin‐treated group displayed a substantial decrease in this proportion, reducing it to a mere 5%, regardless of LPS or RANKL stimulation (Figure 5A–D). Likewise, AO dye, which emits red fluorescence under acidic conditions, is used to detect the acid vesicles of osteoclasts [45]. Notably, there was a significant decline in the red‐to‐green fluorescence intensity ratio among osteoclasts in the Norwogonin group (Figure 5E–H), indicating a repression of acid secretion and bone resorption activity in osteoclasts. Besides, by employing a hydroxyapatite‐coated 48‐well plate, the bone resorption assay further confirmed a significant impairment of bone resorption function in osteoclasts due to the Norwogonin treatment (Figure 5I–L).

### 3.6 Norwogonin Inhibits the Expression of Osteoclastic Marker Genes and Proteins

**FIGURE 6:**
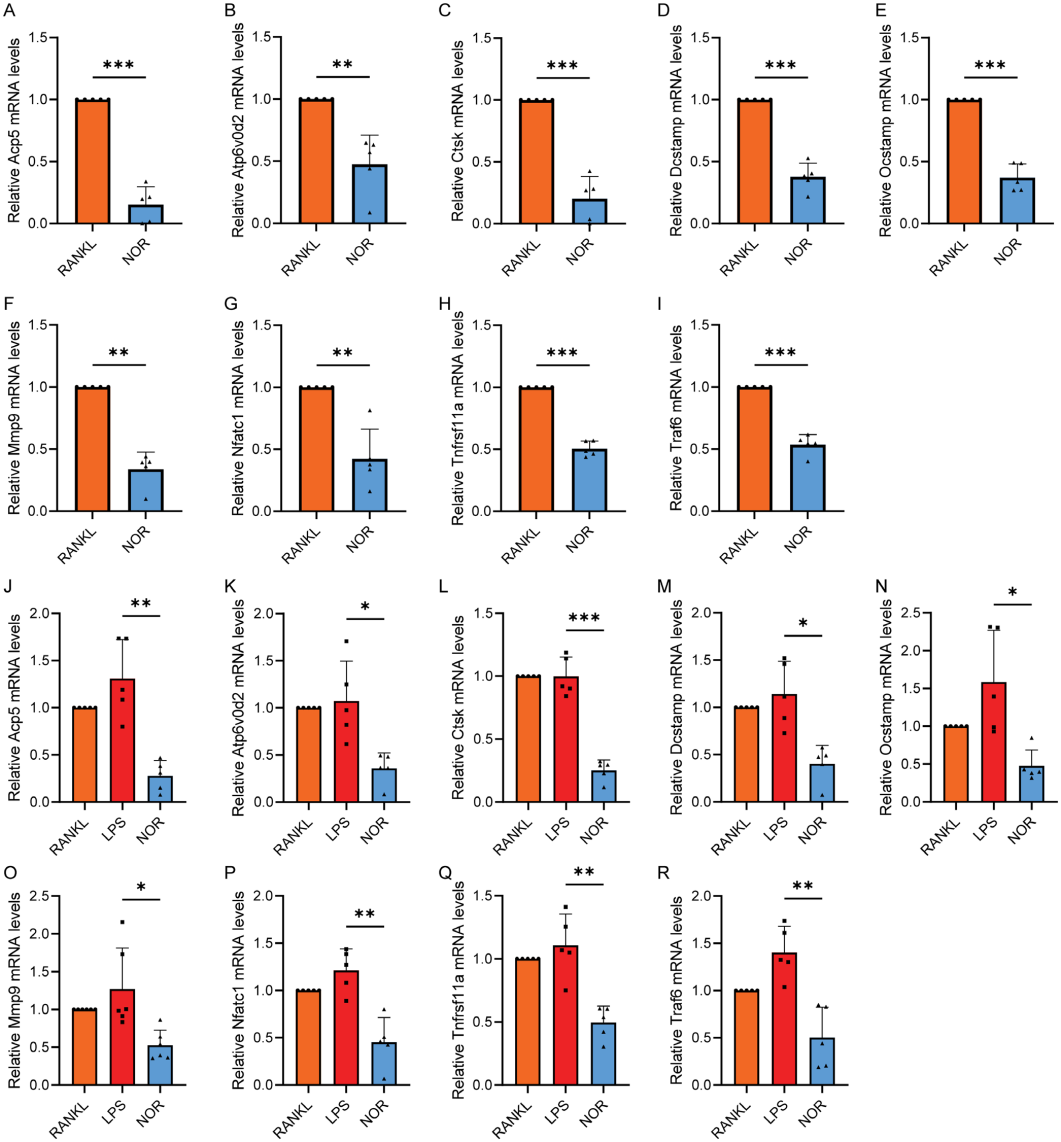
Norwogonin inhibits the expression of osteoclastic marker genes. RT‐qPCR analysis showed mRNA levels of Acp5 (A, J), Atp6v0d2 (B, K), Ctsk (C, L), Dcstamp (D, M), Ocstamp (E, N), Mmp9 (F, O), Nfatc1 (G, P), Tnfrsf11a (H, Q) and Traf6 (I, R). Quantitative results were normalised to Gapdh with a $2^{-\Delta \Delta C_{t}}$ method. All quantitative data were analysed with T‐test or one‐way ANOVA and presented as mean ± SD from five biologically independent experiments. ***p < 0.001, **p < 0.01, *p < 0.05. NOR, Norwogonin.

**FIGURE 7:**
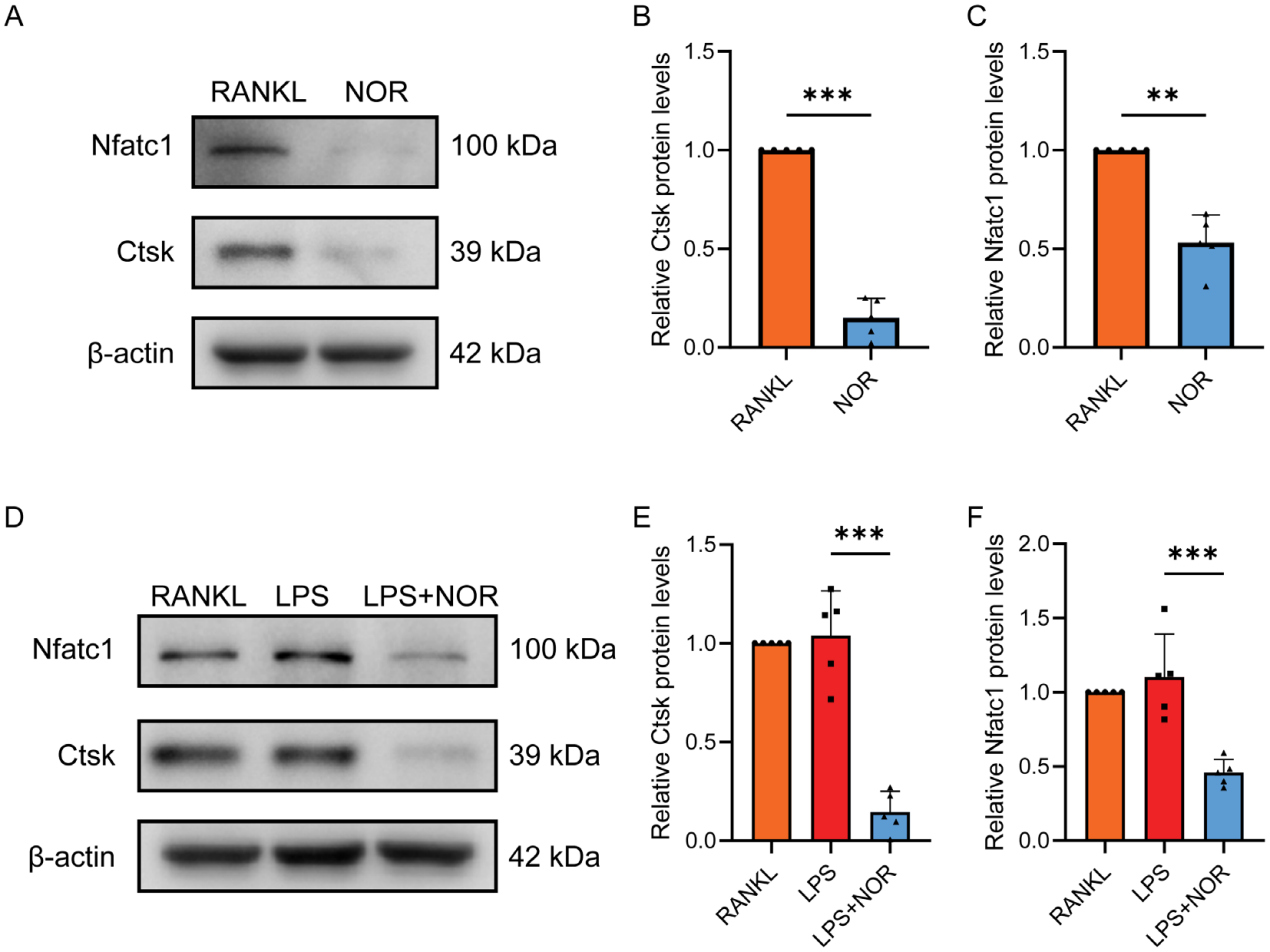
Norwogonin inhibits the expression of osteoclastic marker proteins. (A, D) WB showed the protein expression level of Nfatc1 and Ctsk in osteoclasts post various cultures. The quantitative data were normalised to β‐Actin and are presented as mean ± SD in (B, C, E and F). All quantitative data were analysed with T‐test or one‐way ANOVA and presented as mean ± SD from five biologically independent experiments. ***p < 0.001, **p < 0.01, *p < 0.05. NOR, Norwogonin.

To better unveil the mechanisms responsible for Norwogonin's suppression of LPS‐induced osteoclast formation and function, we assessed the expression of canonical osteoclast markers at both the RNA and protein levels. QPCR analysis revealed that all osteoclast‐specific genes, including those related to cell differentiation (Traf6, Tnfrsf11a and Nfatc1), cell fusion (Atp6v0d2, Dcstamp and Ocstamp) and resorption function (Ctsk, Acp5 and Mmp9), presented equal or higher expression in the LPS group compared with the RANKL group, but were markedly downregulated in the Norwogonin‐treated conditions (Figure 6). We further corroborated these observations through WB at the protein level, which yielded comparable results. A significant suppression of Nfatc1 and Ctsk protein expression was validated under Norwogonin treatment (Figure 7), aligning with our previous observations that Norwogonin inhibited osteoclast formation and function (Figures 4 and 5).

### 3.7 Norwogonin Suppresses LPS‐Induced Osteoclastogenesis Through Mitigating ROS Surge and Ca^2+^ Oscillations

**FIGURE 8:**
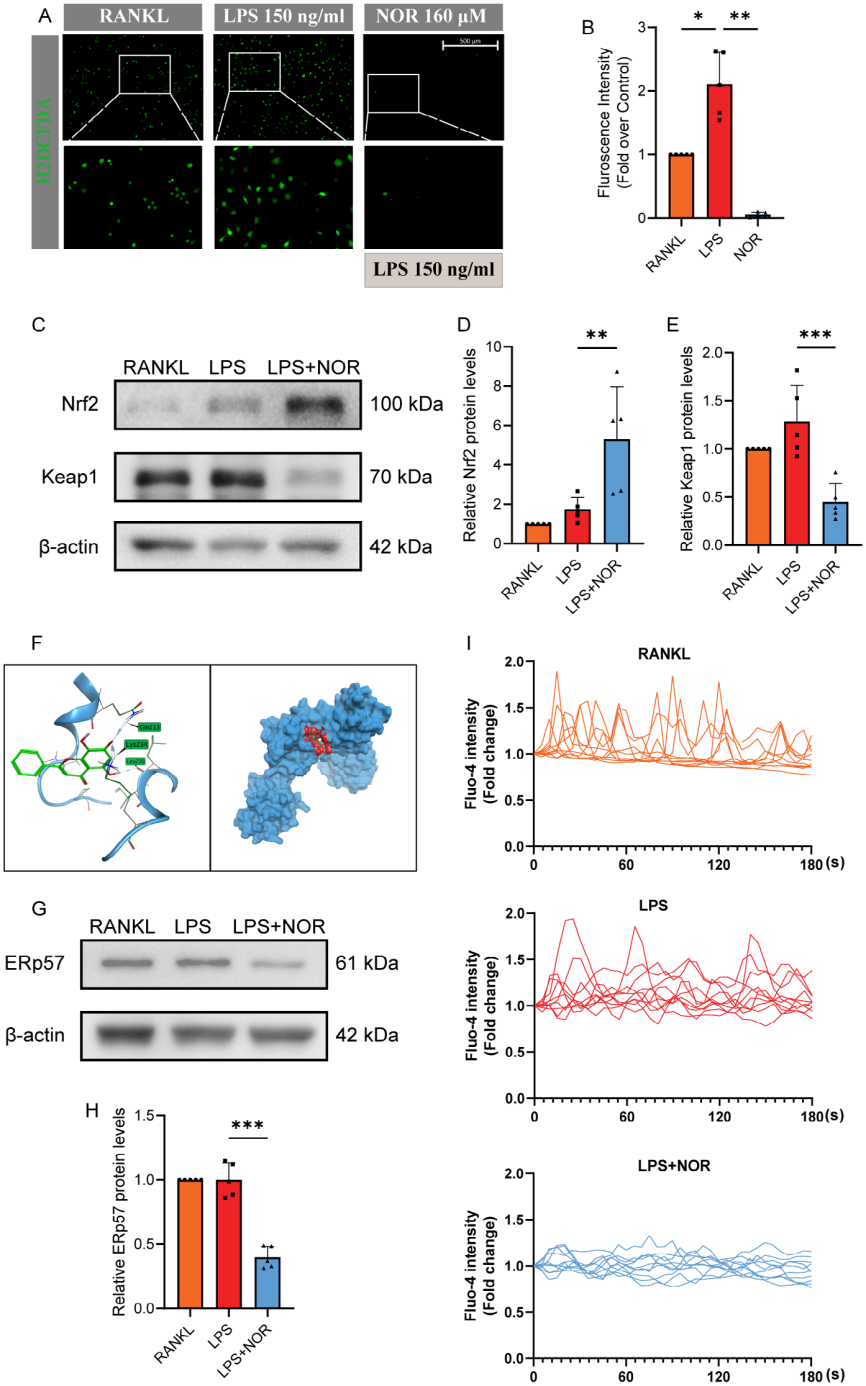
Norwogonin suppresses LPS‐induced osteoclastogenesis through activating ROS scavenger and repressing ROS formation. (A) Intracellular ROS level of osteoclast precursors. Followed by culturing with or without Norwogonin (160 μM) for 24 h, osteoclast precursor cells in each group were treated with DCFH‐DA probe for 20 min and stimulated with RANKL (40 ng/mL), LPS (150 ng/mL), or LPS and Norwogonin (160 μM) for 10 min. Intracellular ROS labelled by DCFH‐DA probe was visualised by a three‐dimensional digital confocal system (EVOS M700, Thermo Fisher Scientific). (B) Quantitative analysis of the fluorescence intensity in (A). (C) WB showed the protein expression level of Nrf2 and Keap1 in the groups treated with RANKL, LPS and Norwogonin. Protein from each group was extracted 4 days after the first RANKL addition. Protein quantification of Nrf2 and Keap1 was normalised to β‐Actin and shown in (D) and (E). (F) Molecular docking of Norwogonin with ERp57. (G, H) Protein expression of ERp57 in osteoclasts treated with RANKL, LPS and Norwogonin. (I) Intracellular Ca^2+^ oscillations of osteoclast precursors. After treating with indicated agents for 3 h in each group, cells were treated with fluo‐4 probe for 40 min and captured under a fluorescent microscope. Quantitative data for western blot and DCFH‐DA fluorescence intensity were analysed with one‐way ANOVA and presented as mean ± SD from five biologically independent experiments. ***p < 0.001, **p < 0.01, *p < 0.05. NOR, Norwogonin.

Since LPS is a classical RA‐mimicking agent that promotes the secretion of inflammatory factors, such as IL‐17A, TNF‐α and IL‐1β [56, 57], and ROS plays a pivotal role as a central mediator in inflammatory pathways and reactions [58], we proceeded to investigate the effects of LPS on the ROS scavenger system, along with unravelling the potential mechanisms by which Norwogonin impairs LPS‐induced osteoclast differentiation and function. In osteoclast precursor cells, after stimulation, the intracellular ROS level was detected using the H_2_DCFDA probe. Compared with the RANKL group, LPS stimulation induced a substantial rise in ROS generation. However, Norwogonin curbed this surge, managing to restrict the ROS level to a considerably lesser quantity (Figure 8A,B). To authenticate the molecular mechanism explaining this phenomenon, we conducted a WB assay to identify variations in the ROS scavenger system. The results revealed that LPS stimulation mildly provoked the expression of both Nrf2 and Keap1 proteins, likely a response to ROS overproduction. Notably, Norwogonin treatment markedly promoted Nrf2 expression but dampened that of Keap1 (Figure 8C–E).

Considering the intimate relationship between ROS signalling and Ca^2+^ oscillation [24, 46], and the crucial role of ERp57‐mediated calcium signalling in osteoclast differentiation as confirmed by our previous research [59], we have also determined their variations under the influence of Norwogonin treatment. From molecular docking, Norwogonin presented effective interaction with the ERp57 protein (Figure 8F). The intracellular Ca^2+^ oscillations, as indicated by the Fluo‐4 fluorescent probe (Figure 8H), were activated following stimulation via either RANKL or LPS, but were significantly reduced by Norwogonin. Consistently, the expression of ERp57 was also suppressed post Norwogonin treatment.

Collectively, these findings attest to Norwogonin's promising capability in fine‐tuning the ROS‐related signalling and ERp57‐mediated Ca^2+^ oscillations, ultimately inhibiting LPS‐induced osteoclast differentiation and function.

### 3.8 Norwogonin Mitigated Inflammatory Bone Destruction and Reduced Osteoclast Numbers in CIA Model

**FIGURE 9:**
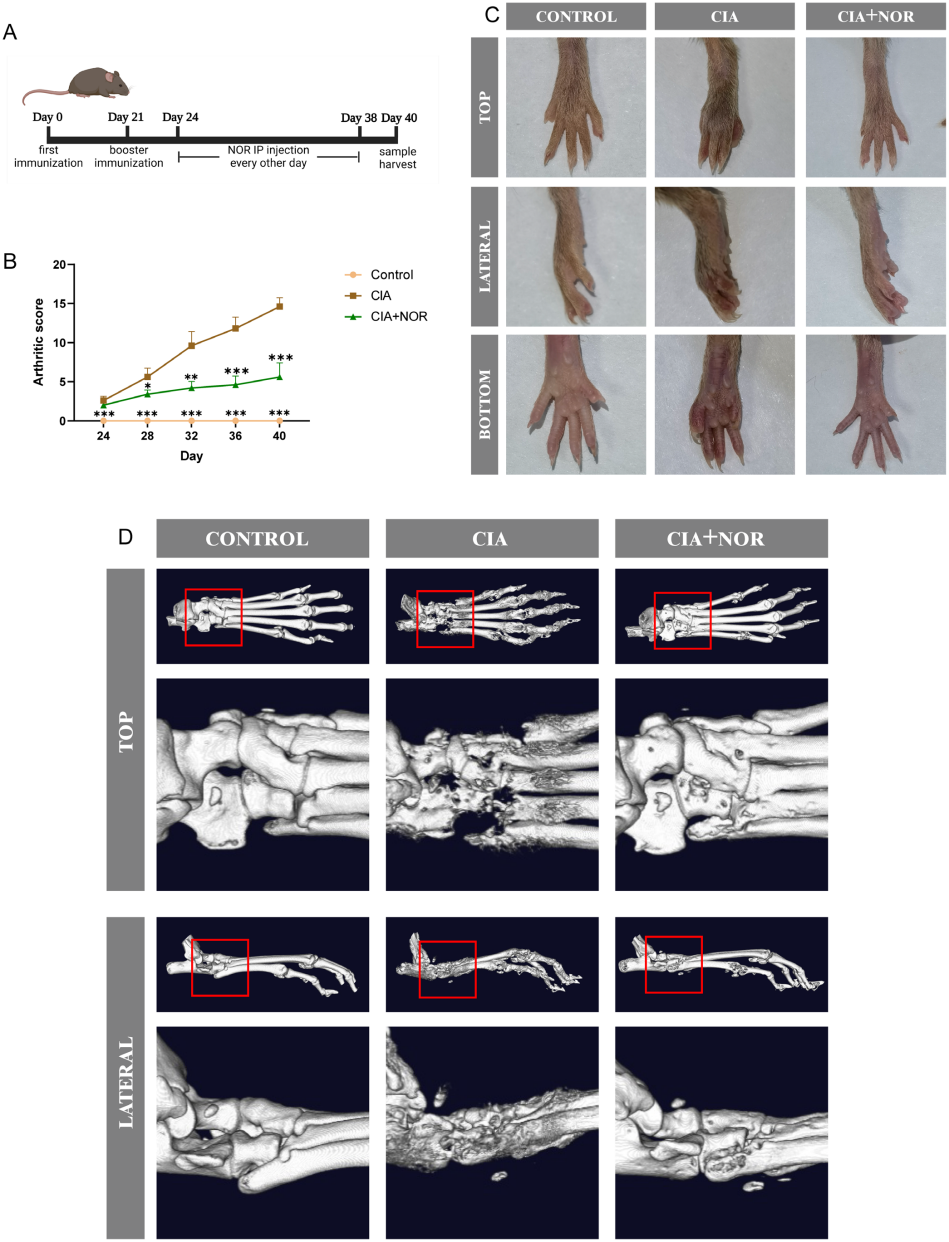
Norwogonin protects against arthritis progression and joint destruction in CIA mice. (A) Schematic diagram of CIA model construction and Norwogonin treatment strategy. (B) Arthritic score from Day 24 to Day 40. The statistical difference of Control and CIA + NOR groups compared with the CIA group was analysed with one‐way ANOVA and presented as mean ± SD. (C) Hind paw of mice in three different groups was photographed on Day 40. (D) Representative three‐dimensional (3D) reconstructed CT images of the ankle and paw joints in the three groups. All animal data were collected from biologically independent quintuplicates. ***p < 0.001, **p < 0.01, *p < 0.05. NOR, Norwogonin.

CIA is a well‐established RA model, which presents similar pathological manifestations and immune progression with RA [48]. Based on the bioinformatic evidence and results obtained from in vitro verification, we further explored the potential effect of Norwogonin on the CIA model. Mice were randomly assigned to the control and CIA groups and injected with Norwogonin (30 mg/kg) or saline every other day through intraperitoneal injection from Day 24 to Day 38 (Figure 9A). Norwogonin treatment successfully mitigated the swelling and redness compared with the CIA group (Figure 9B), and the arthritic score showed a difference of 9 points between the CIA and CIA + Norwogonin groups on Day 40 (Figure 9C). 3D reconstruction of μCT results in paw and ankle joints presented severe bone destruction in the CIA group. However, Norwogonin treatment protected the small joints and prevented disease progression (Figure 9D). Therefore, our in vivo assay further confirmed the therapeutic potential of Norwogonin in mitigating RA‐related bone destruction.

## 4 Discussion

The quest for effective therapeutic agents to alleviate RA articular symptoms and impede disease progression continues to face significant challenges. Norwogonin, a flavonoid compound extracted from *S. baicalensis*, has demonstrated anti‐infectious, anti‐diabetes and anti‐cancer properties in previous studies [33, 34, 60]. In this study, by employing network pharmacological methods, we postulate a correlation between Norwogonin and RA, with a high probability of it being related to the regulation of ROS levels and osteoclast activity under inflammatory conditions. Moreover, as expected, our biological validations provide the first evidence to prove Norwogonin's potent inhibitory effect on osteoclast differentiation and function under LPS stimulation, at both cellular and molecular levels, potentially attributable to the enhancement of the scavenger system and reduction in ROS levels.

The identical targets between Norwogonin and RA were firstly deciphered in this study, aiming to predict the potential pharmacodynamic mechanisms of Norwogonin in RA treatment. Interestingly, 18 shared targets were presented to involve in the regulation of the inflammatory response through protein kinase activity and oxidoreductase activity in the cytoplasm, thereby affecting osteoclast differentiation and functionality during RA. Prior research indicates that osteoclasts, the primary bone‐resorbing cells, are susceptible to various inflammatory factors and elevated ROS levels [61, 62]. This suggests not only that inflammation‐triggered osteoclast hyperactivity is the central mediator and effector in pathological osteolysis of RA, but also that it is a crucial intervention target for preventing or treating inflammatory bone destruction [53]. Flavonoids represent a broad class of natural products known for their remarkable anti‐inflammatory properties, and have thus become an important treatment option for RA [30]. Belonging to the flavonoid family, Norwogonin is expected to possess common characteristics including anti‐inflammatory and even anti‐osteoclast capabilities. Furthermore, core targets associated with Norwogonin possess a high interaction relationship with other protein targets, demonstrate an intimate correlation with inflammatory response and osteoclast differentiation and exhibit excellent docking mode and score with Norwogonin. Therefore, the network pharmacological and molecular docking results imply Norwogonin as a promising candidate for the prevention or treatment of RA‐induced bone erosion.

We further scrutinised the actual effect of Norwogonin on osteoclast differentiation and resorption function under LPS stimulation. LPS is a known inducer of the inflammatory response, triggering inflammatory osteolysis and other disorders [63]. Through activating either surface or intracellular receptors of macrophages and other host cells, LPS can initiate the inflammasome‐related signalling pathway, bolstering production of inflammatory factors, such as IL‐6, 1 L‐8. IL‐1β and TNFα, thus facilitating the activity of osteoclast lineage cells [64]. In addition, another study demonstrated the direct activation of osteoclast differentiation by LPS via the toll‐like receptor 4 (TLR4) axis [65]. Meanwhile, previous research also established that LPS stimulation alone could not induce osteoclast differentiation from primary BMMs. Instead, it was a prerequisite that the initial fusion process of preosteoclasts required RANKL stimulation, enabling the inflammatory factors secreted by macrophages under LPS stimulation to maintain subsequent osteoclast maturation and function, irrespective of RANKL withdrawal after the cell fusion stage [55]. We found corroborating results in our study as well, demonstrating that LPS stimulated osteoclast differentiation and survival in a dose‐dependent manner upon RANKL removal, and a comparable effect of RANKL was observed with 150 ng/mL of LPS. Nonetheless, Norwogonin treatment led to a significant decrease in the formation of mature osteoclasts, regardless of LPS or RANKL stimulation. Consistent results were also found in the osteolytic function assays. The formation of F‐actin rings is essential for osteoclast adhesion and motility, which also creates a locally sealed zone for the secretion of inner acidic vesicles, thereby enabling bone resorption [66]. Our study confirmed that while F‐actin ring and acidic compartment formation were similar under RANKL or LPS stimulation, they were predominately repressed by Norwogonin. Consistently, the resorption function of the osteoclast was completely inhibited under Norwogonin treatment. Therefore, these phenotypic results suggest that Norwogonin could potentially serve as an efficient inhibitory agent for osteoclast activities. Therefore, these phenotypic results suggest Norwogonin could potentially serve as an effective inhibitory agent in osteoclast activities.

The changes in cell behaviours are typically regulated by the alterations in the expression of key genes and proteins. Our research validated that Norwogonin inhibited the expression of osteoclast‐specific genes involved in cell differentiation (Traf6, Tnfrsf11a and Nfatc1), cell fusion (Atp6v0d2, Dcstamp and Ocstamp) and cell function (MMP9, Ctsk and Acp5) under both LPS and RANKL stimulation. We further analysed the expression of Nfatc1 and Ctsk, fundamental mediators in osteoclast differentiation and functionality, using WB. The results were consistent with those obtained from the qPCR assay. Therefore, both at the transcriptional and translational levels, we affirmed the inhibitory effect of Norwogonin on osteoclast differentiation and function induced by either LPS or RANKL.

ROS plays a key role in facilitating cellular activities within the osteoclast. Primarily, ROS serves as an integral component for signalling transduction in osteoclast differentiation. It functions as a second messenger downstream of RANKL activation, regulating subsequent signalling pathways [50, 67, 68, 69, 70]. Moreover, under inflammatory conditions, osteoclast‐generated ROS overproduction may exacerbate the local inflammatory milieu, disrupting local metabolic balance and further promoting RANKL expression by fibroblasts and lymphocytes [71, 72, 73]. Concurrently, Nrf2, a key figure in the ROS scavenger system and typically suppressed due to Keap1 binding, gets activated and translocated into the nucleus due to exposure to stressors or inducers, subsequently dominating the expression of antioxidant enzymes [74, 75]. Our study corroborated these findings, where LPS stimulation significantly increased the ROS level compared with the RANKL group. The expression of Nrf2 and Keap1 also experienced a mild increase, indicating a responsive activation of the cellular antioxidant system due to excessive ROS formation under LPS‐induced inflammatory conditions as reported [76, 77]. While a previous study has highlighted the antioxidant property of Norwogonin in relieving oxidative stress in PC12 cells under hypoxic conditions, the underlying molecular mechanism remains to be elucidated [78].

**FIGURE 10:**
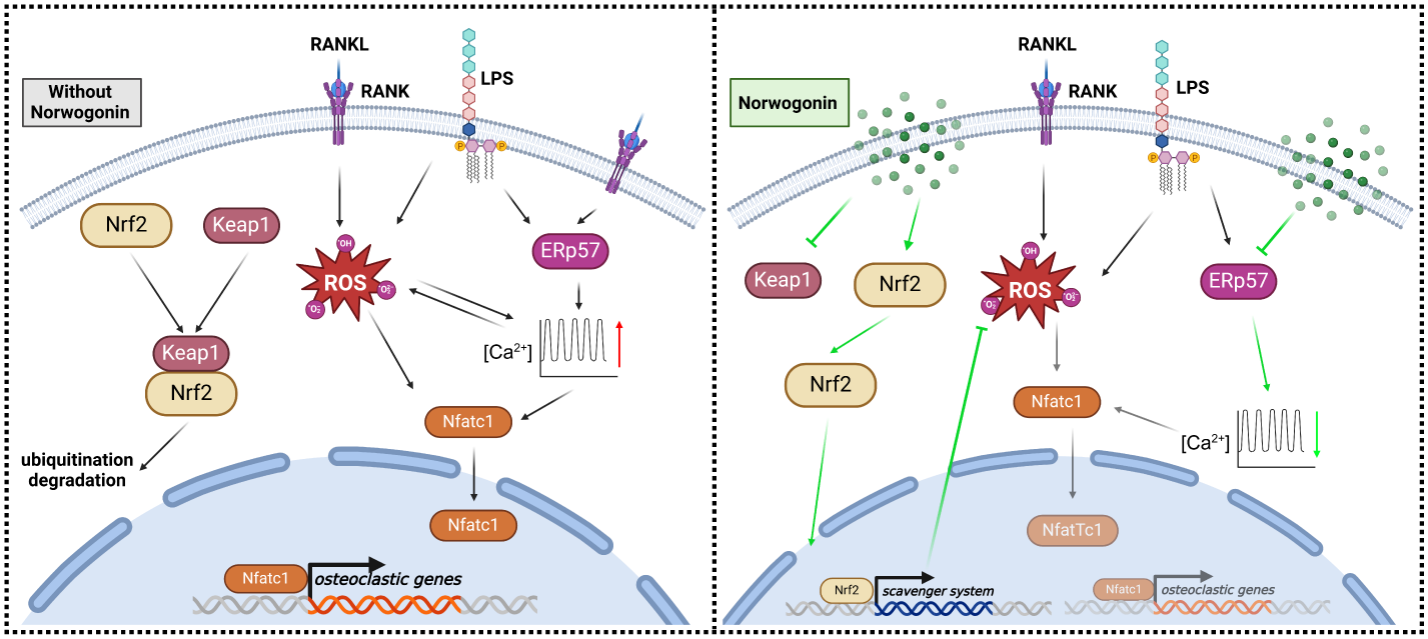
Potential signalling pathways of Norwogonin working on osteoclast. LPS can stimulate both osteoclast differentiation and function by promoting the RANKL/RANK‐ROS axis, which triggers the downstream transcription factor Nfatc1 and subsequently activates osteoclastic gene expression. Conversely, Norwogonin fosters Nrf2 expression while inhibiting Keap1 expression, thereby attenuating LPS‐induced redox imbalance and subsequent cellular behaviours of inflammatory osteoclasts (Draft of Figure 10 was created with BioRender.com).

Serving as a molecular chaperone with oxidoreductase activity, ERp57 plays a pivotal role in regulating intracellular Ca^2+^ concentration and ROS level [59, 79]. Furthermore, the intricate relationship between Ca^2+^ oscillations and ROS signalling influences the behaviours of LPS‐stimulated osteoclasts [46]. In this study, we not only validated the inhibitory effect of Norwogonin on ERp57 expression, but also observed its mitigating impact on LPS‐induced Ca^2+^ oscillations. Collectively, our study demonstrated that the treatment with Norwogonin under LPS stimulation successfully augments Nrf2 expression while inhibiting the expression of Keap1 and ERp57. This leads to a significant reduction in ROS levels and Ca^2+^ oscillations within osteoclast precursor cells (Figure 10).

Although the results in this study have raised evidence to support the inhibitory effect of Norwogonin on osteoclastogenesis through mitigating oxidative stress and regulating calcium signalling, we will further scrutinise the uncharted effect of Norwogonin and precise targets in the future. Significantly, through establishing ERp57 conditional knockout or overexpression models, we aim to unravel the interaction between ERp57 and Norwogonin, and the ramifications of Norwogonin under different levels of ERp57, thus underscoring ERp57 as a major target of Norwogonin in the osteoclast lineage.

Meanwhile, considering the complexity of RA pathogenesis and the involvement of multiple immune cell lineages, a more comprehensive investigation of the effects of Norwogonin on these cells is also necessary to thoroughly evaluate its therapeutic potential. However, related research remains lacking in the current study. Therefore, we plan to integrate omics methods, including transcriptomics and proteomics, to reveal multiple signalling pathways and identify potential treatment targets, ultimately establishing a comprehensive and precise mechanistic framework of Norwogonin.

In vivo assays can offer stronger evidence during treatment‐effect verification. Therefore, the CIA model was established to verify the true effect of Norwogonin in RA condition. In our study, Norwogonin successfully alleviated joint erythema and swelling, and retarded bone destruction by reducing osteoclast numbers in CIA mice. Therefore, the consistent results yielded from biological analyses, in vitro assays and in vivo experiments collectively indicate the therapeutic potential of Norwogonin in alleviating inflammatory osteolysis in RA.

## 5 Conclusions

Based on the network pharmacology and biological validation, our study demonstrated the therapeutic effect of Norwogonin on LPS‐stimulated osteoclast differentiation and function through activation of the antioxidant system and inhibition of Ca^2+^ oscillations. The CIA‐related arthritic condition and bone destruction were also alleviated by Norwogonin treatment. Our findings provide beneficial evidence for the therapeutic application of Norwogonin in the prevention and treatment of RA‐related inflammatory osteolysis.

## Author Contributions

**Haojue Wang:** data curation (lead), formal analysis (lead), resources (lead), software (lead), writing – original draft (lead). **Tao Yuan:** investigation (equal), validation (equal), visualization (equal), writing – original draft (equal). **Xiao Yu:** data curation (equal). **Yi Wang:** data curation (equal), investigation (equal). **Changxing Liu:** conceptualization (equal), supervision (equal). **Ziqing Li:** funding acquisition (equal), writing – review and editing (equal). **Shui Sun:** funding acquisition (equal).

## Ethics Statement

All experiments involving mice were performed following the protocol approved by the Institutional Animal Care and Use Committee (IACUC) of the Shandong Provincial Hospital Affiliated to Shandong First Medical University (Shandong, China; No. LS2023047).

## Conflicts of Interest

The authors declare no conflicts of interest.

## Supporting information


**Figure S1.** Flow cytometry analysis of isolated bone‐marrow‐derived macrophages.

**Table S1.** All constituents of *Scutellaria baicalensis* retrieved from TCMSP database.

**Table S2.** Related targets of Norwogonin retrieved from TCMSP, Herb and SwissTarget database.

**Table S3.** Related targets of RA retrieved from CTD and DisGeNET database.

**Table S4.** Results of GO and KEGG enrichment analysis retrieved from David database.

**Table S5.** Results of PPI network analysis retrieved from String database.

## Data Availability

The data that support the findings of this study are available from the corresponding author upon reasonable request.
