# Supplementary material for: Norwogonin Attenuates Inflammatory Osteolysis and Collagen‐Induced Arthritis via Modulating Redox Signalling and Calcium Oscillations
Source: J Cell Mol Med. 2025 Mar 18;29(6):e70492. doi: 10.1111/jcmm.70492 (PMC11915625; doi:10.1111/jcmm.70492)
Supplement: Supplementary file 1 — Figure S1. Flow cytometry analysis of isolated bone‐marrow‐derived macrophages. [file JCMM-29-e70492-s003.docx]

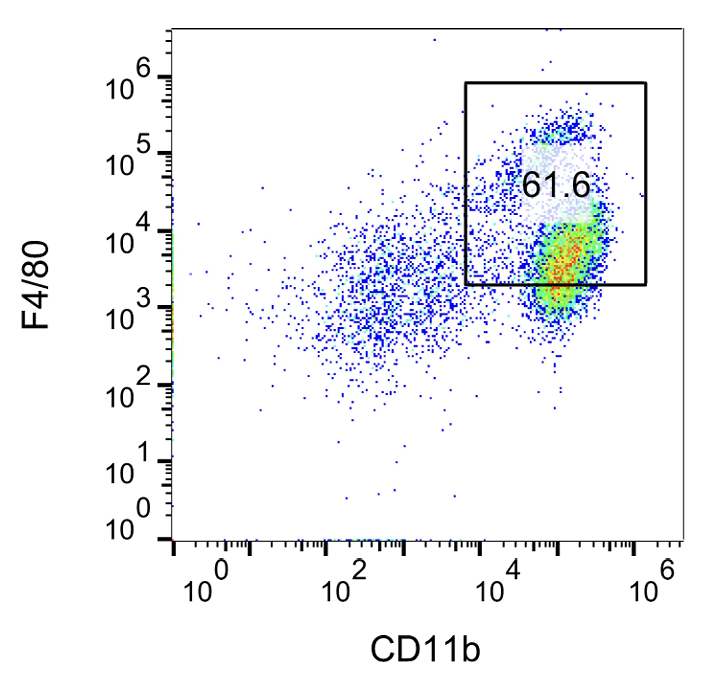

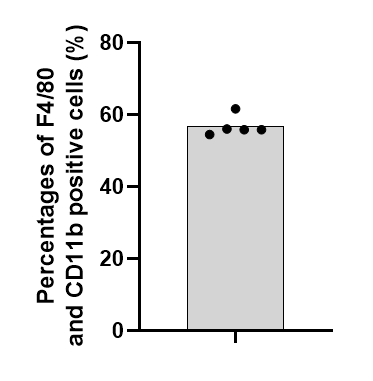


Supplemental figure 1. Flow cytometry of isolated Bone marrow-derived macrophages proved 60% of total cells were double positive for F4/80 and CD11b.
